# A follow-up study on the effects of an educational intervention against pharmaceutical promotion

**DOI:** 10.1371/journal.pone.0240713

**Published:** 2020-10-28

**Authors:** M. Murat Civaner

**Affiliations:** Department of Medical Ethics and History of Medicine, Bursa Uludag University School of Medicine, Bursa, Turkey; University of Toronto, CANADA

## Abstract

**Background:**

The promotion strategies of pharmaceutical companies create many problems including irrational prescribing, diminished trust in the patient-physician relationship and unnecessary increases in pharmaceutical costs. Educating prescribers is known to be one of the few potentially effective measures to counteract those impacts. However such educational programs are limited in the literature, and their effectiveness against the effects of hidden curriculum in the long term is unknown. This study aims to evaluate the effectiveness of an education program both in the short term and the long term after the students have been exposed to informal and hidden curriculum and various pharmaceutical promotion methods.

**Methods:**

A longitudinal and controlled study was carried out in a school of medicine in Turkey where there are no restrictive policies for pharmaceutical promotion. A survey was applied to 123 students who attended the class throughout the terms of 2011–12, 2012–13, and 2013–14, evaluating the pre-educational status of students’ opinions of promotion and any post-educational changes. A follow-up study four years later asked those three cohorts to fill out the same survey to see the possible effects of the clinical environment and various promotion methods. Also, the opinions of all 518 sixth-year students who had not taken the class in those three terms were compared to the educated students.

**Results:**

The program was significantly effective in the short term in changing students’ opinions and attitudes positively towards recognizing companies’ discourse and promotion strategies. But in the long term, the education lost its ability to convince students of the importance of not getting financial support for scientific activities from pharmaceutical companies (p:0.006) and carrying out research (p<0.001). In addition, although the educated students were more aware that trivial gifts could influence prescriptions compared to the uneducated 6th year students (p<0.001), the difference between them and the uneducated students generally becomes less significant when they encounter the clinical environment. The study also evaluated students highly-exposed to promotion; for this sub-group, the educated students kept their consciousness level about the influences of trivial gifts (p<0.001) while the uneducated students were confident that they were immune to the influence of trivial gifts.

**Conclusions:**

The education program could be used for creating awareness of, increasing skepticism towards, and inculcating disapproval about pharmaceutical promotion practices. However, the effectiveness of the educational intervention is susceptible to erosion after exposure to the informal and hidden curriculum together with exposure to promotion. The impact of role-models, organizational culture, and institutional policies could be important aspects to be addressed for sustaining the effectiveness of such education programs.

## Introduction

### Definition of the problem

The pharmaceutical market is growing every year [1], and total pharmaceutical revenues worldwide exceeded one trillion U.S. dollars for the first time in 2014 [2]. The pharmaceutical industry generates higher profit margins than any other industry [3,4]. On the other hand, faced with worldwide competition, economic volatility, increasing costs, patent restrictions and mass production of generics, while only a small percentage of new drugs are an advance [5], promotion has become particularly important for pharmaceutical companies (PCs), and promotional spending exceeds research & development spending [6]. This huge push of promotion strategies creates many problems including irrational prescribing since the information they provide could be one-sided, incorrect, or even deceptive [7–9]. WHO states one of the important reasons for the irrational use of medicine as “inappropriate promotion of medicines and profit motives from selling medicines” [10]. A comprehensive review concludes that there is a strong association between reliance on promotion on the part of PCs and inappropriate use of prescription drugs [7]. In addition to this substantial risk to public health, it has been shown that promotional methods might also erode professional values and demean the profession, diminish trust in the patient-physician relationship, and increase costs unnecessarily [7,11–13]. Prescription practices are influenced by promotional methods since the ads are prepared by sophisticated professional advertising and public relations companies, whereas independent resources for continuous medical education are often non-existent, and infrastructural shortages create opportunities for PCs to establish gift-relations [14]. The other reason prescription practices are susceptible to pharmaceutical promotion is the lack of education of prescribers; they are uneducated both on interpreting scientific data [15,16], and on pharmaceutical advertising strategies [17]. Also, physicians usually do not believe that their prescribing behaviors are influenced by promotion methods [7,13,18,19], which makes the problem harder to deal with.

### Tackling the problem

According to a comprehensive review, the industry’s self-regulation, supervision of journal editors, guidelines for advertising and sales representatives, and post-marketing regulations are ineffective interventions for preventing/decreasing the problems related to the promotional methods of pharmaceutical companies (PCs) [7]. The review found that the only potentially effective measures are official regulations implemented by governments, researching and disclosing deceptive promotion strategies, and educating prescribers about promotion methods. Therefore, training medical students and physicians about the promotional methods of PCs seems crucial if their opinions and attitudes are to be changed and their support enlisted in counteracting the negative impact of pharmaceutical promotion. The American Medical Student Association, Healthy Skepticism. Inc., No Free Lunch, and PharmAware recommend four objectives for educational initiatives regarding pharmaceutical and device promotion [12]. All health professionals should be “educated explicitly about decision making and evaluation of evidence and promotion, helped to understand that there is no proven method for enabling them to gain more benefit than harm from promotion, helped to understand their responsibility to avoid pharmaceutical and device promotion, and educated explicitly about the most reliable sources of information”. On the other hand, a world-wide study covering 64 countries concluded that the lack of importance of drug promotion in medical education stands in stark contrast to the large volume of promotion strategies targeting health professionals [20].

### What is not known

The number of educational interventions that aim to teach trainees how to cope with the promotion strategies of PCs is limited [21–32]. Most are in the format of short lectures and seminars lasting 1 hour to 1 day, and their effectiveness was assessed only by pre-post evaluations without following-up those cohorts after they proceed to clinical years and without comparison to a control group. A systematic review dated 2017 concluded that there are no data available on the sustainability of the effects of such courses on participants’ behavior [33]. Moreover, if there are no policies for restricting promotion in their learning environments, students in the clinical years are exposed to two confounding influences: they are frequently exposed to pharmaceutical promotion, and the extent of their contact with PCs is associated with positive attitudes about promotion and skepticism about any negative implications of those interactions [34]. Students are also exposed to "an unscripted, predominantly ad hoc, and highly interpersonal form of teaching and learning that takes place among and between faculty and students (the informal curriculum), and a set of influences that function at the level of organizational structure and culture (the hidden curriculum)" [35]. Features of learning environments, including institutional policies and role models, have an important influence on professional identity development [36]. Students’ opinions and attitudes about promotional activities could be shaped by both informal and hidden curriculum (IC&HC) during clinical years, as it was reported by final-year students that they base their drug choice mainly on examples provided by their medical teachers [37]. Therefore, as one study reviewing the impact of these education programs concludes, “It is not entirely clear from these studies that the changes in attitudes and/or behavior are sustainable over the long run. (…) More research is needed to determine the long-term impact of these educational interventions” [38]. To this end, a study was carried out to evaluate the effectiveness of an educational program about promotional strategies. It specifically aimed to evaluate the program’s durability during clinical years when students encounter the full force of the influences that prevail where there is no specific regulation for preventing exposure to promotion.

## Materials and methods

### The intervention

An educational program titled “Physician-Healthcare Industry Interactions” was developed by the author, aiming to help future-physicians create awareness, develop a “healthy skepticism” [39], and inculcate disapproval about the promotional methods of PCs. The program was structured based on a literature review of the promotion methods of PCs, how they are applied, how they can negatively affect the prescriptions of physicians, the arguments of physicians for and against pharmaceutical relations, and what can be done to prevent the effects of promotional methods. The author’s studies on the types of promotion strategies and physicians’ arguments for and against relationships with PCs were among the main resources of the basis of the program [14,40–42]. Accordingly, the main goals of the program were defined as supporting medical students to think critically on promotional methods and pro and con arguments about promotion, to teach individual measures to cope with the negative influences of promotion, and to recognize the limitedness of individual measures (the impossibility of “dancing with porcupine” [43] without getting hurt). The educational program, tailored to those goals, formulated the following curriculum:

- The types of interactions with the healthcare industry
  - Rights and responsibilities of different parties
  - Related legal regulations and professional codes
- The nature and motives of a pharmaceutical company as an entity
  - Marketing as a scientific discipline
  - Types of promotional methods and how they operate
- The influential effects of promotional methods on clinical decisions
- Soundness of the arguments for and against physician-PCs interactions
- Protection from negative impacts of PC promotion methods
  - Individual measures
    - Rational prescribing
    - Reaching out to independent scientific resources
    - Avoiding promotion methods
  - Institutional measures

The program was implemented as a 14 hour elective to the 123 2^nd^ year medical students who selected the program throughout the terms of 2011–12, 2012–13, and 2013–14 in Bursa Uludag University School of Medicine (UUSM), Turkey, where there is no institutional policy implemented for restricting pharmaceutical promotion. PC representatives can promote their drugs in the hospital, give gifts, drug samples, and promotional materials to physicians. Also, PCs can donate materials necessary for the continuation of routine service in the hospital, they can provide financial support for the participation and organization of scientific meetings, and for conducting scientific research.

The topics were delivered both by classical lectures and by educational methods such as small interactive group sessions. The lectures on promotion were given by a professional from a company specializing in marketing education. Group activities included critical appraisal of promotional materials for the validity of claims, critical analysis of covert drug advertising in newspapers and on the internet to elicit implicit/subliminal messages, writing regulations from scratch then comparing and critically appraising the current ones, using case scenarios to analyze arguments for and against physician-PCs relationships. Students also engaged in role-play with a real PC sales representative, followed by detailed explanations about how certain strategies are used to promote pharmaceutical products. As another interactive method, students were shown a shortened version of a movie titled “Side Effects” [44] about the professional life of a pharmaceutical representative and asked to spot the promotion methods; the ways of implementing those methods were then discussed.

### Evaluating the effectiveness

A survey form was developed by the author, consisting of six background questions and ten statements. The background questions established gender, whether they took the elective and any other education about physicians-healthcare industry relationships, opinions about the program, and exposure to direct and indirect promotion methods. The first group of statements presented the claims about the nature and necessity of PCs and asked students to agree or disagree, including factual errors and faulty inferences gathered from various sources [41,45–51]. The second group of statements, gathered from the literature [42], were devised to reflect the optimal professional mindset that maintains a healthy skepticism about pharmaceutical promotion to which students responded with their opinions and attitudes. The survey form was pilot tested on 30 2^nd^ year medical students who had not taken the class and revised accordingly. Its internal consistency was moderate (Cronbach’s alpha: 0.623).

The effectiveness of the program was evaluated in a two-phase study, aiming to measure it in the short term (after the class), and the long term (four years later in the 6^th^ year of medical education). In Phase I, the survey was applied to a total of 123 students who attended the class in three consecutive terms, 2011–12, 2012–13, and 2013–14, evaluating the pre-educational status of students’ opinions and any post-educational changes in students’ opinions.

The Phase II evaluation aimed to evaluate the effectiveness of the program in the last year of medical education, hypothesizing that the hidden curriculum and different kinds of promotion activities might have a negative impact on students’ approach to interactions with the industry even after having a special training in their preclinical years. Therefore, a follow-up study four years later asked those three cohorts to fill out the same survey for comparison in 2015–16, 2016–17, and 2017–18 terms when they became 6^th^ year students.

In addition, 518 6^th^ year students who had not taken the “Physician-Healthcare Industry Interactions” class in their 2^nd^ year of medical education in the same terms were also enrolled into the Phase II study in the terms of 2015–16, 2016–17, and 2017–18 as the control group and asked to complete the same survey. Their opinions were compared to the students who had attended the program as the intervention group to evaluate the program’s effectiveness via assessment of outcomes in the intervention and control groups.

**Fig 1:**
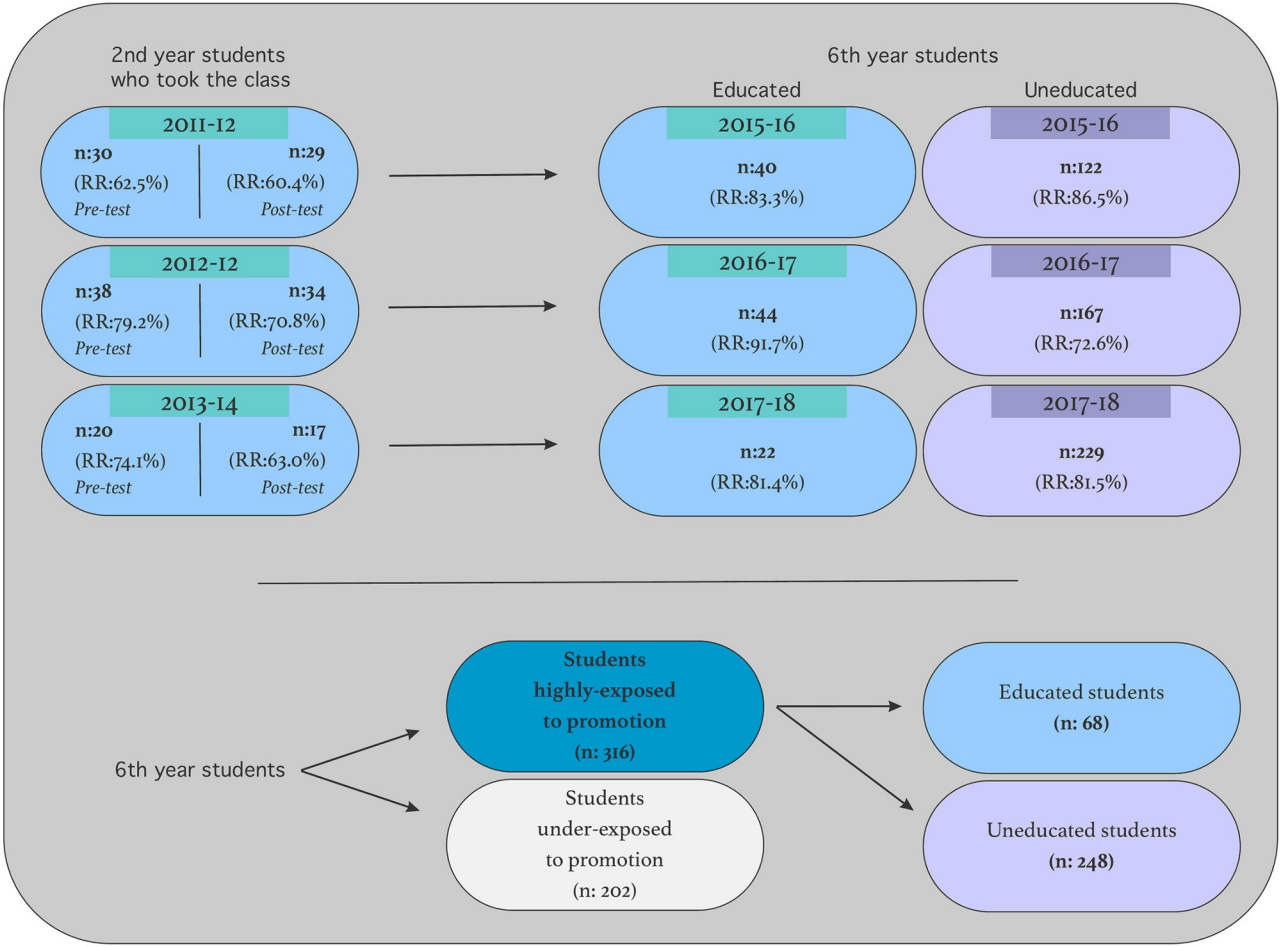
The study design. (RR: Response rate).

One final comparison sought to determine whether the education program is protective against the effects of high exposure to various promotional methods in the clinical years, as it is known that there is a positive association between exposure and immunity belief [7,12,18,19,52,53]. To that aim, all 6^th^ year students were asked to report their exposure to 15 promotional methods they had witnessed or been directly exposed to throughout the clinical rotations. These included 11 promotional methods they might have witnessed such as gifts given to a physician or observation of a representative’s detailing (considered as indirect exposure and scored 1 point for each) and four methods that were applied directly to students (considered as direct exposure and scored 2 points for each). A total of 19 points could have been scored. Of all 6^th^ year students, 316 had an exposure score of 10 or more and were accepted as ‘highly-exposed’ to promotion. Then, in this sub-group of highly-exposed 6^th^ year students, results from the survey were used to compare the opinions of 68 students who attended the class to 248 students who had not. The study design is shown in Fig 1, together with response rates.

### Statistical analysis

Students were asked to express their level of agreement on a three-point Likert scale (Agree/Not sure/Disagree). The level of agreement to the statements on the discourse of the pharmaceutical industry and promotional methods was scored as 1, 2, and 3 for ‘Agree’, ‘Not sure’, ‘Disagree’, respectively. The statements expressing a critical position was scored as 3, 2, and 1 for ‘Agree’, ‘Not sure’, ‘Disagree’, respectively. The median scores and interquartile ranges for each item were calculated to analyze the possible significant differences between compared groups. Wilcoxon signed-rank test was used for comparing median scores before and after the educational intervention. Mann-Whitney U test was used for comparing median scores of the cohorts in 2^nd^ and 6^th^ year, educated and uneducated students both in the 6^th^ year and in the highly-exposed group. All statistical comparisons with a p-value below 0.05 were assumed statistically significant. Statistical analyses were performed with IBM SPSS Statistics 23.0.

The number of students in the intervention group (123) was the total number of the participants attending the class throughout the three terms. In other words, 123 students were not selected for the study; they were the entire population of students who took the elective course. Therefore no sample size was calculated. Those 123 students were the intervention group to understand the short term and long term effectiveness of the class. Nevertheless a post-hoc power analysis was done. The item #8 (“Trivial gifts such as pens or mugs given by pharmaceutical companies cannot influence prescriptions.”) was chosen for the power analysis since it reflects one of the main learning objectives of the educational intervention which aims to convince the students otherwise. Post-hoc power was calculated as 0.69 for the comparison of the students opinion right after they had the class and when they become 6^th^ year students four years later. For the comparison of 6th year students who took the class and who did not, post-hoc power was calculated as 0.99 for the item #8 (The analysis were made by G-Power v. 3.1.9.2).

Institutional permission was granted from the Deanery of the UUSM, and the UUSM Research Ethics Board approved the study as ethically justifiable (June 30, 2015; #2015-13/11). All participants were informed both verbally and in writing about the aim and nature of the study, their right not to participate or to quit the study when they would like to, that not participating would not effect their education in any way, and the data would be collected anonymously.

### Patient and public involvement

Patients and members of the public were not directly involved in this study.

## Results

Of 635 students participating in the study, 50.2% (n:317) were female. One out of every five students had taken the elective class in 2^nd^ year (19.2%; n:120), and only 3.9% (n:25) stated that they had taken a short lecture on the issue during medical ethics or public health classes. Most of the students who took the class stated that the program was generally useful (89.2%; n:107), and it was helpful in making medical decisions more objective and unbiased (80.8%; n:97).

After participating in the “Physician-Healthcare Industry Interactions” class, students changed their opinions significantly as measured by their pre- and post- education surveys (Table 1). Post-education, they moved from ‘Agree’ to ‘Not sure’ for the claim of *“If companies did not support R&D studies*, *many of the current drugs would not have been developed” (p*:*0*.*003)*, and they agreed more that promotional methods should be within certain limits *(p*:*0*.*028)*. The education program also effectively created awareness that they were not capable of fully mitigating the effects of promotional methods on their prescribing practices *(p*:*0*.*02)*. Post-education, the students disagreed that trivial gifts such as pens or mugs cannot influence prescriptions *(p<0*.*001)*, they were not sure about getting financial support from PCs for organizing and participating in scientific activities *(p<0*.*001)* and meeting with representatives of PCs *(p*:*0*.*001)*, and they were significantly more opposed to PC-sponsored research *(p<0*.*001)*.

**Table 1:** Comparison of pre- and post- education for the 2^nd^ year students.

		Pre-Test Median (IQR)	Post-Test Median (IQR)	p
*On the nature and necessity of PCs*	Scientific developments in medicine require huge investments and the money needed can only be afforded by private companies, not the state.	2 (1–3)	3 (1–3)	0.737
	If companies did not support R&D studies, many of the current drugs would not have been developed.	1 (1–2)	2 (1–3)	**0.003**
	Drugs should be a commercial commodity just like the other commodities in the market which are bought and sold.	3 (3–3)	3 (3–3)	0.107
	It is normal that, similar to other sectors, pharmaceutical companies give priority to increasing their profits or they cannot afford to develop new drugs.	2 (1–3)	1 (1–3)	0.082
	Pharmaceutical promotion should be conducted within certain limits.	1 (1–1)	1 (1–1)	**0.028**
*Opinions and attitudes about promotion*	I don’t think that I am competent enough to cope with marketing methods.	1 (1–2)	1 (1–3)	**0.020**
	I think positively about getting financial support from the companies for organizing and participating in scientific activities since they contribute to physicians’ scientific development.	1 (1–1)	2 (1–3)	**<0.001**
	Trivial gifts such as pens or mugs given by PCs cannot influence prescriptions.	1 (1–1)	3 (2–3)	**<0.001**
	Carrying out research with the financial support of PCs may create pressure on researchers, therefore I am against PC-sponsored research.	2 (2–3)	1 (1–2)	**<0.001**
	I think physicians should deny meeting with representatives of PCs.	3 (2–3)	2 (1–3)	**0.001**

While the pre-and post-education measures soundly showed that the education program positively affected student knowledge and professionalization in the short-term, the longitudinal effects of the program have a different outlook. When the cohorts who took the class became 6^th^ year students four years later, they rejected the claim *“It is normal that*, *similar to other sectors*, *pharmaceutical companies give priority to increasing their profits or they cannot afford to develop new drugs” (p*:*0*.*006*), and they kept their critical position on the nature and necessity of PCs they had four years ago (Table 2). Also, they were even more sure in their last year of education that trivial gifts could influence prescriptions *(p*:*0*.*022)*, and agreed that “I think physicians should deny meeting with representatives of PCs.” *(p<0*.*001)*. On the other hand, they were significantly more in agreement with getting financial support from PCs for organizing and participating in scientific activities *(p*:*0*.*006)*, and for carrying out research *(p<0*.*001)*. Besides, despite the statistical non-significance, changes in median scores show that students were more in agreement with the statement *“If companies did not support R&D studies*, *many of the current drugs would not have been developed”*, and they were feeling relatively more competent about coping with promotional activities, compared to their opinions in 2^nd^ year.

**Table 2:** Comparison of the post-test results of 2^nd^ year students to the 6^th^ year results of the same cohorts.

		2^nd^ year Post-Test Median (IQR)	6^th^ year Median (IQR)	p
*On the nature and necessity of PCs*	Scientific developments in medicine require huge investments and the money needed can only be afforded by private companies, not the state.	3 (1–3)	3 (1–3)	0.347
	If companies did not support R&D studies, many of the current drugs would not have been developed.	2 (1–3)	1 (1–2)	0.061
	Drugs should be a commercial commodity just like the other commodities in the market which are bought and sold.	3 (3–3)	3 (3–3)	0.196
	It is normal that, similar to other sectors, pharmaceutical companies give priority to increasing their profits or they cannot afford to develop new drugs.	1 (1–3)	3 (1–3)	**0.006**
	Pharmaceutical promotion should be conducted within certain limits.	1 (1–1)	1 (1–1)	0.107
*Opinions and attitudes about promotion*	I don’t think that I am competent enough to cope with marketing methods.	1 (1–3)	2 (1–3)	0.376
	I think positively about getting financial support from the companies for organizing and participating in scientific activities since they contribute to physicians’ scientific development.	2 (1–3)	1 (1–3)	**0.006**
	Trivial gifts such as pens or mugs given by PCs cannot influence prescriptions.	3 (2–3)	3 (1–3)	**0.022**
	Carrying out research with the financial support of PCs may create pressure on researchers, therefore I am against PC-sponsored research.	1 (1–2)	3 (1–3)	**<0.001**
	I think physicians should deny meeting with representatives of PCs.	2 (1–3)	1 (1–1)	**<0.001**

As for the comparison of the intervention and the control group of 6^th^ year students, there was no difference between the two groups regarding the statements on the nature and necessity of PCs; all statements were disapproved by both groups (p>0.05), except the statement of *“If companies did not support R&D studies*, *many of the current drugs would not have been developed”* (Table 3). These findings were the same also for the group of students highly-exposed to promotion methods, showing that the students, educated or not, were usually skeptical of PCs’ discourses even under high-exposure to promotion (Table 4).

**Table 3:** Comparison between the 6th year students who took the class and who did not.

		Did not take the class Median (IQR)	Took the class Media (IQR)	p
*On the nature and necessity of PCs*	Scientific developments in medicine require huge investments and the money needed can only be afforded by private companies, not the state.	3 (1–3)	3 (1–3)	0.488
	If companies did not support R&D studies, many of the current drugs would not have been developed.	1 (1–2)	1 (1–2)	0.920
	Drugs should be a commercial commodity just like the other commodities in the market which are bought and sold.	3 (3–3)	3 (3–3)	0.179
	It is normal that, similar to other sectors, pharmaceutical companies give priority to increasing their profits or they cannot afford to develop new drugs.	3 (1–3)	3 (1–3)	0.264
	Pharmaceutical promotion should be conducted within certain limits.	1 (1–1)	1 (1–1)	0.830
*Opinions and attitudes about promotion*	I don’t think that I am competent enough to cope with marketing methods.	1 (1–2)	2 (1–3)	**<0.001**
	I think positively about getting financial support from the companies for organizing and participating in scientific activities since they contribute to physicians’ scientific development.	1 (1–2)	1 (1–3)	0.093
	Trivial gifts such as pens or mugs given by PCs cannot influence prescriptions.	1 (1–3)	3 (1–3)	**<0.001**
	Carrying out research with the financial support of PCs may create pressure on researchers, therefore I am against PC-sponsored research.	2 (1–3)	1 (1–3)	0.256
	I think physicians should deny meeting with representatives of PCs.	3 (2–3)	3 (3–3)	0.249

**Table 4:** Comparison between the 6^th^ year students who took the class and who did not, in the highly-exposed to promotion group.

		Did not take the class Median (IQR)	Took the class Median (IQR)	p
*On the nature and necessity of PCs*	Scientific developments in medicine require huge investments and the money needed can only be afforded by private companies, not the state.	3 (1–3)	3 (1.25–3)	0.201
	If companies did not support R&D studies, many of the current drugs would not have been developed.	1 (1–2)	1 (1–3)	0.459
	Drugs should be a commercial commodity just like the other commodities in the market which are bought and sold.	3 (3–3)	3 (3–3)	0.053
	It is normal that, similar to other sectors, pharmaceutical companies give priority to increasing their profits or they cannot afford to develop new drugs.	3 (1–3)	3 (1–3)	0.959
	Pharmaceutical promotion should be conducted within certain limits.	1 (1–1)	1 (1–1)	0.702
*Opinions and attitudes about promotion*	I don’t think that I am competent enough to cope with marketing methods.	1 (1–3)	3 (1–3)	**0.001**
	I think positively about getting financial support from the companies for organizing and participating in scientific activities since they contribute to physicians’ scientific development.	1 (1–2)	1 (1–3)	0.309
	Trivial gifts such as pens or mugs given by PCs cannot influence prescriptions.	1 (1–2)	3 (1–3)	**<0.001**
	Carrying out research with the financial support of PCs may create pressure on researchers, therefore I am against PC-sponsored research.	1 (1–3)	1 (1–3)	0.900
	I think physicians should deny meeting with representatives of PCs.	3 (3–3)	3 (3–3)	0.831

Regarding students’ opinions and attitudes towards PCs’ promotion methods, the education program created a significant difference between the intervention and the control group on the opinions about the effects of trivial gifts. Among all 6^th^ year students and also in the highly-exposed group (50.6% of all 6^th^ year students), the educated students were more aware that trivial gifts could influence prescriptions, while the uneducated ones were confident that those kind of gifts could not be influential *(p<0*.*001 for both groups)*. The educated students in the 6^th^ year, and also in the highly-exposed group, were against PC-sponsored research; while the uneducated students agreed more with them when they were highly exposed to promotion.

On the other hand, contrary to the education’s aim, the educated students were feeling more confident about their skills to cope with promotional methods *(p<0*.*001 in 6*^*th*^ *year group; p*:*0*.*001 in the highly-exposed group)*. Also, both educated and uneducated students took a similar affirmative stance on the statements about getting financial support from PCs for scientific activities and meeting with representatives are positively viewed *(p>0*.*05)*.

## Discussion

### Findings in context

Comprehensive researches show that educating physicians and medical students is one of the few potentially effective measures for addressing the negative impacts of pharmaceutical promotion [7]. Yet studies of educational interventions on the issue are limited in the medical education literature. Based on this fact, an education program was developed for 2^nd^ year medical students in UUSM and shown by pre-post evaluation to be effective for taking a critical stance against self-promoting discourse of PCs and becoming more skeptical of promotion strategies.

A positive change right after an educational intervention is not unexpected as the other 11 studies in the literature showed a positive change of knowledge and attitude by pre-post evaluations of specific education programs [21–31]. However, the durability of those programs’ effectiveness is not clear in clinical years, where students connect to healthcare intensely, observe patient-healthcare worker relationships in a real setting, and begin to comprehend healthcare provision in all its dimensions. In this regard, this study followed longitudinally the three cohorts who took the class in the 2^nd^ year of their education and measured the durability of effectiveness four years later after they had clinical rotations. It found that the effectiveness of the program was mostly enduring for the statements designed to measure the students’ opinions on the nature and necessity of PCs. Even, while 2^nd^ year students mostly thought that PCs needed to prioritize profits to afford the development of new drugs despite the educational intervention, they significantly disagreed with that as of 6^th^ year students, became more professionally robust. This picture is the same when they are compared to uneducated students, and also when they are compared to those under the influence of high-exposure to promotion methods. A possible explanation could be that the students had that opinion even before they started their medical training. Another possibility is that medical students over the clinical years, educated or not, develop a skepticism about the rationales of the absolute necessity of PCs for scientific developments, the need for prioritizing profits over people, and the promotion of drugs as any other commodity. This kind of consciousness might have been developed by realizing how drug prices are determined in the market, lack of access to the care needed -especially drugs, and the obstacles to a right to health. A specific education focused on PCs’ promotion could contribute to the development of that consciousness in an earlier phase, which might be important for gaining a more patient-centered view for future-physicians. Apart from these positive changes, education lost its positive effect on the 6th year student’s opinions about getting financial support from PCs for scientific activities and carrying out research, and also on the statement “If companies did not support R&D studies, many of the current drugs would not have been developed”, a loss which could be connected to the effects of promotion and the informal and hidden curriculums.

Apart from following longitudinally the cohorts who participated in the educational program, this study took the 6^th^ year students who had not participated in the program as the control group, a benchmark against which the effectiveness of the program in the long term could be measured. Change and differences between compared groups regarding the opinions and attitudes on promotional methods are more complex. The effects of the education are consistently positive in compared groups on the influence of trivial gifts and PC-sponsored research. The educated students kept their consciousness level both in the 6^th^ year and in the highly-exposed group that trivial gifts could influence prescriptions, while the uneducated students were confident of the converse. This is concordant with studies in the literature showing that high exposure is associated with more affirmative judgments about the relationships with PCs and more confidence that physicians are immune to promotion methods [19,42,52–54]. This finding shows that the education program has a clear protective effect against an overinflated sense of immunity to pharmaceutical promotion even under high exposure to the promotional methods and even in contradistinction to the IC&HC. The program’s effectiveness endured regarding the opinions about PC-sponsored research as well, and notably, the uneducated students in the highly-exposed group agreed that they were against PC-sponsored research.

However, the students became more approving of getting financial support from pharma for organizing and participating in scientific activities, and less opposed to meeting with pharma representatives when they encountered the clinical environment. It means that the education lost its effectiveness against some very influential promotion methods, failing to help students “understand that there is no proven method for enabling them to gain more benefit than harm from promotion”, one of the recommended objectives for such programs [12]. Being exposed to direct and indirect promotion strategies, and also to the influence of informal and hidden curriculum might be the major factors for explaining this pattern. Medical students’ professional identity is formed in this context both by formal education and also by the hidden curriculum [55]. Factors such as value atmosphere, organizational culture, and behaviors of role-models all can have an influence on attitudes [56], since students internalize structural imperatives and the attitudes and behavioral models in the clinical setting [57]. Therefore the attitudes of clinicians towards promotional activities, their discourses, and adopted norms in daily routine might be conveyed to the students in this regard. The other finding that supports this explanation is the discrepancy between student’s opinions on the nature and necessity of PCs and their attitudes towards PCs’ promotional activities. They usually kept a critical stance in regard to statements claiming that PCs are absolutely needed for drug development, that PCs need to prioritize profits, and that drugs should be a commercial commodity like any others—possibly by recognizing a right to access to healthcare according to need. Nevertheless, they could not make the connection between those claims they rejected and PC promotional activities and failed to see the whole picture. This is a point where theoretical knowledge and abstract values clash with the practical world in the clinics, which they have no idea of beforehand. Therefore any educational intervention (and medical education in general) should aim to make students adopt those values thoroughly and understand their implications in actual healthcare settings before they face the clinical environment. Also, effectiveness could be increased by repeating similar programs in clinical years together with clinicians, since moral education of the students cannot be provided solely by ethicists, as Haferty and Franks emphasized: “The overall process of medical education is presented as a form of moral training of which formal instruction in ethics constitutes only one small piece” [36]. Education could be combined with training on communication skills and rational prescribing. That kind of education could help students see themselves, the clinical environment, and the nature of promotion in more nuanced ways. More importantly, restricting the exposure of the students to promotional strategies by institutional policies could create a significant impact to increase the endurance and effectiveness of such educational initiatives, since it is known that such policies can make future physicians immune to the persuasive aspects of promotion [58–60].

### Strengths and limitations of the study

The main strength of the study is its methodology since the effectiveness of the educational intervention was evaluated longitudinally for the first time by following cohorts and comparing them to a control group after they had been exposed to the clinical environment. One of the limitations of the study is that ‘effectiveness’ was evaluated by a non-validated survey based on the opinions and attitudes of students, rather than by observing their behaviors towards pharmaceutical promotion and their prescriptions after graduation. The study was conducted at one medical school, so the results may not necessarily be generalizable to other academic medical centers. The students may have answered the post-intervention survey in a socially desirable manner. Also, it was not possible to match individual educated students’ replies since they forgot their nicknames when they became 6^th^ year students. Not investigating the 2^nd^ year students who had not taken the class posed another limitation for evaluating their opinions when they become 6^th^ year students. The quantitative nature of the study is another limitation since it was not possible to inquire into the elements of the IC&HC and how they interact with the process of changing or stabilizing students’ opinions.

### Further studies

Educating students will not be sufficient in the long term unless the negative influence of elements of the IC&HC, i.e. the organizational culture and the attitudes of role models, are illuminated with further researches. Studies using qualitative methodologies are needed for gaining a deeper insight into the influential dynamics and factors in the clinic, considering the HC conceptual map suggested by Hafferty and Castellani [61]. A practical method called REVIEW, suggested by Mulder et al, could be used to that purpose by facilitating reflection and discussion on the HC by faculty members and trainees [62]. It could be useful to understand how students’ judgments are shaped and how education loses its effectiveness when there are no restrictions on pharmaceutical promotion.

Qualitative researches are also needed in order to explain unexpected results. It is known that preclinical and clinical students were less likely to feel sufficiently educated on the topic of physician–pharmaceutical industry interactions [34]. However, the cohorts followed with this study felt gradually more competent to cope with promotion strategies and this difference became more apparent in the highly-exposed group. Since one of the aims of the education program was to create an awareness that one could never be totally immune to the influence of promotion, the reasons for this transformation of confidence needs further research to determine whether this transformation is a positive effect of the education (i.e. by giving reasonable confidence against the promotion strategies) or a failure that could be connected to the interactions in clinics.

## Conclusions

There is an important gap in the medical curricula for preparing future physicians to interact with the pharmaceutical industry. As was shown by this study, it is possible to claim that the education program developed in UUSM is effective and protective against promotion strategies, and could be used for creating awareness of, increasing skepticism towards, and inculcating disapproval about promotional activities. However, this study also found that even an effective educational program can lose its durability in the clinical environment.

Therefore the impact of role-models, organizational culture, and practical experiences could be important aspects to be addressed in the curriculum to sustain the effectiveness of such education programs. More importantly, minimizing the exposure to pharmaceutical promotion could also be a significant intervention that would increase the durability of such educational initiatives, since individual protection provided by education can be effective only to a certain degree and getting hurt by promotion is more or less inevitable when “dancing with the porcupine”. In addition to institutional policies of prohibiting promotion, alternative information sources that are unbiased, freely accessible, and updated should be available to all students and physicians if pharmaceutical promotion is to be banned.

## Supporting information

S1 Dataset (ZIP)

S1 Survey form (PDF)

S1 Course syllabus (DOCX)
