# Supplementary material for: A follow-up study on the effects of an educational intervention against pharmaceutical promotion
Source: PLoS One. 2020 Oct 28;15(10):e0240713. doi: 10.1371/journal.pone.0240713 (PMC7592808; doi:10.1371/journal.pone.0240713)
Supplement: S1 Survey form — (PDF) [file pone.0240713.s002.pdf]

COURSE SYLLABUS

ENGLISH		
**1**	**Course title**	Physician-Healthcare Industry Interactions
**2**	**Type of course**	Elective
**3**	**Level of course**	Undergraduate
**4**	**Year of study**	2
**5**	**Semester**	2
**6**	**Hour/week**	1
**7**	**Prerequisites**	None
**8**	**Language**	Turkish
**9**	**Mode of delivery**	• Classical lectures<br>• Small interactive group sessions<br>• Role-playing
**10**	**Contact information of the Course coordinator**	Prof.Dr. M. Murat Civaner<br>mcivaner@gmail.com / Tel: +90.224.295 4272<br>Address: Uludag Universitesi Tip Fakultesi, Temel Tip Bilimleri Binası zemin kat, Tip Tarihi ve Etik AD, Gorukle, 16059, Bursa, Turkey
**11**	**Website**	deontoloji.uludag.edu.tr

**12**	**Objective of the course**	To create awareness about marketing methods and their effects, to generate an ability to think critically about related arguments, to devise individual measures to cope with the negative influences of pharma-marketing, and to recognize the limitedness of individual measures.
**13**	**Course content**	

		**COURSE CONTENT**
	Week	
	1	Setting the stage: Basic concepts and data from the field
	2	The different parties of physician–healthcare industry relationships and their responsibilities
	3	Marketing and Advertisement as a scientific discipline
	4	Company as an entity: Its features and obligations
	5	Marketing methods of PCs - I
	6	Marketing methods PCs - II
	7	Drug rep's detailing techniques
	8	Drug promotion in audio-visual media
	9	Disease mongering
	10	The impacts of marketing methods
	11	Related regulations
	12	Analysis of the arguments on physician–healthcare industry relationships
	13	Analysis of the arguments on physician–healthcare industry relationships
	14	What can be done for prescribing rationally?

14	**Textbooks, references and/or other materials**
	Elliot C. Beyaz Önlük, Siyah Şapka. Hayy Kitap, 2012, İstanbul.<br><br>Moynihan R. Satılık Hastalıklar. Hayy Kitap, 2006, İstanbul.<br><br>Goldacre B. Kötü İlaç. Pegasus yayınevi, 2016, İstanbul.<br><br>Moncrieff J. İlaçla Tedavi Efsanesi: Psikiyatrik İlaç Kullanımına Eleştirel Bir Bakış. Metis yayınları, 2009, İstanbul.<br><br>Ellison S. Bir Masalmış Kolesterol. Hayy Kitap, 2007, İstanbul.<br><br>Hailey A. Acı Reçete. E yayınları, 1985, İstanbul.<br><br>Kapitalizm Sağlığa Zararlıdır. HayyKitap, 2013, İstanbul.<br><br>Ellison S. Batı Tıbbı Sağlığınızın Altını Nasıl Oyar? Hayy Kitap, 2008, İstanbul.<br><br>Wlch HG. Aşırı Teşhis – Sağlık Adına Hasta Etmek. İnsev yayınları, 2013, İstanbul.
**15**	**Assesment**

**TERM LEARNING ACTIVITIES**	**NUMBER**	**WEIGHT**
Midterm Exam	1	25
Group work-project	5	25
Final Exam	1	50
Total	7	100
Contribution of Term (Year) Learning Activities to Success Grade		50
Contribution of Final Exam to Success Grade		50
Total		100
