# Supplementary material for: A follow-up study on the effects of an educational intervention against pharmaceutical promotion
Source: PLoS One. 2020 Oct 28;15(10):e0240713. doi: 10.1371/journal.pone.0240713 (PMC7592808; doi:10.1371/journal.pone.0240713)
Supplement: S1 Course syllabus — (DOCX) [file pone.0240713.s003.docx]

**SURVEY FORM**

**Please mark the appropriate choice for the information below:**

1. Gender F M
2. I took the elective class “Physician-Healthcare Industry Interactions” when I was a second-year student.

Yes No

1. *(If you took the class)* The programme was useful for me.

Agree Not sure Disagree

1. *(If you took the class)* I think that programme helps make my medical decisions more objective and unbiased.

Agree Not sure Disagree

1. I received any other education about physicians-healthcare industry interactions during my medical education.

Yes (……………….) No

1. Please mark the statements below if valid for you during clinical rotations *(applied only to the sixth-year students)*

I observed a pharmaceutical company representative (PCR) detailing a drug to a physician.

The PCR gave a sample to the physician during that meeting.

The PCR gave monographs and/or articles to the physician during that meeting.

The PCR gave gifts to the physician during that meeting.

The PCR gave monographs and/or articles to me as well.

The PCR gave gifts to me as well.

I observed physicians attending a drug presentation meeting organized by a PC.

I also attended that drug presentation meeting.

PCs organized drug presentation meetings for sixth-year students and I attended those meetings.

There were drug advertisements in scientific journals in the clinics.

There were drug advertisements on some objects in the clinics.

Some equipment in the clinics was donated by PCs.

A PCR gave financial support to a physician for covering the cost of attending a congress (including travel, accommodation and registration fee).

A PCR proposed financial support to a physician for carrying out clinical research on the patients she cared for.

I have seen a PCR give a medical device or other equipment as a gift for use in daily clinical practice.

**Please mark your level of agreement to the statements below:**

*Statements on the nature and necessity of PCs*

1. Scientific developments in medicine require huge investments and the money needed can only be afforded by private companies, not the state.

Agree Not sure Disagree

1. If companies did not support R&D studies, many of the current drugs would not have been developed.

Agree Not sure Disagree

1. Drugs should be a commercial commodity just like the other commodities in the market which are bought and sold.

Agree Not sure Disagree

1. It is normal that, similar to other sectors, pharmaceutical companies give priority to increasing their profits or they cannot afford to develop new drugs.

Agree Not sure Disagree

1. Pharmaceutical promotion should be conducted within certain limits.

Agree Not sure Disagree

*Opinions and attitudes about promotion methods of PCs*

1. I don’t think that I am competent enough to cope with promotion methods.

Agree Not sure Disagree

1. I think positively about getting financial support from the companies for organizing and participating scientific activities, since they contribute to physicians’ scientific development.

Agree Not sure Disagree

1. Trivial gifts such as pens or mugs given by pharmaceutical companies cannot influence prescriptions.

Agree Not sure Disagree

1. Carrying out research with the financial support of companies may create pressure on researchers, therefore I am against PC-sponsored researches.

Agree Not sure Disagree

1. I think physicians should refuse to meet with representatives of pharmaceutical companies.

Agree Not sure Disagree
